# The Godspeed Questionnaire Series in the Assessment of the Social Robot TIAGo by Older Individuals

**DOI:** 10.3390/s23167251

**Published:** 2023-08-18

**Authors:** Slawomir Tobis, Joanna Piasek-Skupna, Aleksandra Suwalska

**Affiliations:** 1 Department of Occupational Therapy, Poznan University of Medical Sciences, 60-781 Poznan, Poland; stobis@ump.edu.pl; 2 Institute of Robotics and Machine Intelligence, Poznan University of Technology, 60-965 Poznan, Poland; joanna.piasek@put.poznan.pl; 3 Chair and Department of Palliative Medicine, Poznan University of Medical Sciences, 61-245 Poznan, Poland; 4 Department of Mental Health, Chair of Psychiatry, Poznan University of Medical Sciences, 60-572 Poznan, Poland

**Keywords:** humanoid robot, social robot, robot perception, robot acceptance, psychometric properties

## Abstract

(1) Background: A robot in care for older adults requires solid research confirming its acceptance. The aim of this study was to present the Polish version of the Godspeed Questionnaire Series (GQS) and assess the perception of the social robot TIAGo; (2) Methods: The study involved older individuals living in the community and care homes and measured perception after interaction with TIAGo using five series of GQS (S1: Anthropomorphism, S2: Animacy, S3: Likeability, S4: Perceived intelligence, and S5: Perceived safety); (3) Results: We studied 178 individuals (age: 75.2 ± 9.6 years, 103 women). Good internal consistency was found. Cronbach’s Alpha was 0.90 for the entire tool (from 0.75 to 0.94 for the individual series). Mean scores for S1 and S2 were comparable but lower than all others (*p* < 0.001). Average scores for S3 and S4 did not differ but were higher than those of S5. Age, gender and education did not impact the answers, as did the ease of use of technology and self-assessment of independence. Solely, the place of residence influenced the results of S3 and S5; people living in institutions scored higher (*p* < 0.05 and *p* < 0.001, respectively); (4) Conclusions: Acceptance does not go hand in hand with the perception of anthropomorphism and animacy.

## 1. Introduction

In the course of rapid ageing in contemporary societies, more and more attention is paid to the way care for older adults is organised and provided. Among the most important problems that need to be addressed in this area, there is the shortage of care staff and, as a result, an excessive burden on caregivers that may lead to frustration and burnout. An additional factor results from the fact that many older people live alone or with their spouses (being, in most cases, also older persons themselves), which entails specific care requirements. These should also be carefully considered, particularly so in the context of limited support possibilities from formal and informal carers due to their insufficient availability [1]. Possible solutions, including those using smart technologies and robots, that support the contemporary paradigm of ageing in place are thus discussed [2,3]. Ageing in place is an important approach to dealing with limitations of older adults—that are the more likely to arise the more the former progress on the time axis—by ensuring that they are able to stay in their own homes as long as possible [4,5]. This can be accomplished, among others, by performing a periodical evaluation of the needs and abilities of the ageing persons and introducing appropriate assistive technologies to help them cope with emerging problems. These technologies include socially assistive robots [6].

According to the literature review by Andtfolk et al. [7], the use of humanoid social robots is considered in four domains, all of which are essential for maintaining the independence of ageing people: (1) Supports everyday life, (2) Provides interaction, (3) Facilitates cognitive training and (4) Facilitates physical training. Moreover, the prospect of creating joint care teams based on cooperation between humans and robots is even discussed [8]. Henceforth, the aim of the introduction of technology is not to displace people from care or threaten their jobs but to support them, particularly where a shortage of human resources is expected [9]. Furthermore, human caregivers can and should be effective mediators between modern technologies and older adults [10] and also can play a significant role in co-designing new technologies for Ageing in place [11]. This approach, which is relatively new in the discussed age group, also termed “participatory design”, aims to engage the end-user as a partner in design, explicitly including the ideation of new concepts.

Still, the implementation of a robot into care requires solid research confirming not only its effectiveness but also acceptance. As it is impossible to imagine good acceptance without a positive perception of the robot, studying it is crucial. Among the tools created specifically to assess perception are the Negative Attitude toward Robots Scale (NARS), with two subscales, developed by Nomura et al. [12], the Negative Attitudes toward Robots that Display Human Traits (NARHT) and the Negative Attitudes toward Interactions with Robots (NATIR). Notably, all these tools focus on the characteristics of negative experiences. A different approach is taken by the Godspeed Questionnaire Series (GQS) presented by Bartneck et al. [13]. Importantly, this tool is available in many languages, which indicates broad interest in its use [14]. As demonstrated, it is frequently used in HRI research, therein most often in the areas of tourism and education [13]. The majority of published studies analysing the GQS were performed with the use of the NAO robot, for which the GQS scores on the Perceived intelligence scale depended on the role that the robot was supposed to play [13]. NAO in the role of a doctor was perceived as more intelligent than in the role of a patient [15]. The NAO is a small humanoid social robot—its height is just over 0.5 m. Its perception is, therefore, significantly different from that of larger humanoid robots like the TIAGo.

The aim of this paper is to present the Polish version of the Godspeed Questionnaire Series and its application to assess the perception of the social robot TIAGo (PAL Robotics, Barcelona, Spain) in the context of its possible use in the care of older adults.

## 2. Materials and Methods

The study group consisted of older individuals (at least 60 years of age) living in care homes in the Greater Poland (Wielkopolska) region and in the community. The latter were participants of day care centres (run by the city of Poznan), which potentially indicated the existence of limitations in independence and needs related to life activities. In the current study, which included a presentation of the TIAGo robot and the possibility of real-world interaction with it, demographic data (including age, gender, education, place of living and ease of use of technology) as well as measures of perception of the robot using the GQS were collected. The details of the robot’s presentation and interaction were described previously [16]. The meetings started with a presentation of the basics of the project, followed by a brief introduction to TIAGo and its functions. The next step involved interaction with the humanoid social robot, lasting approximately 90 to 150 min—until all interested persons had sufficient opportunity to operate the robot. Eventually, the participants completed the GQS, following their experience with the robot.

The study was verified by the Bioethics Committee of Poznan University of Medical Sciences (Protocol No. 711/18). All studied subjects gave their consent for participation after receiving a full explanation of the nature of the study.

### 2.1. The Translation Procedure

**Figure 1:**
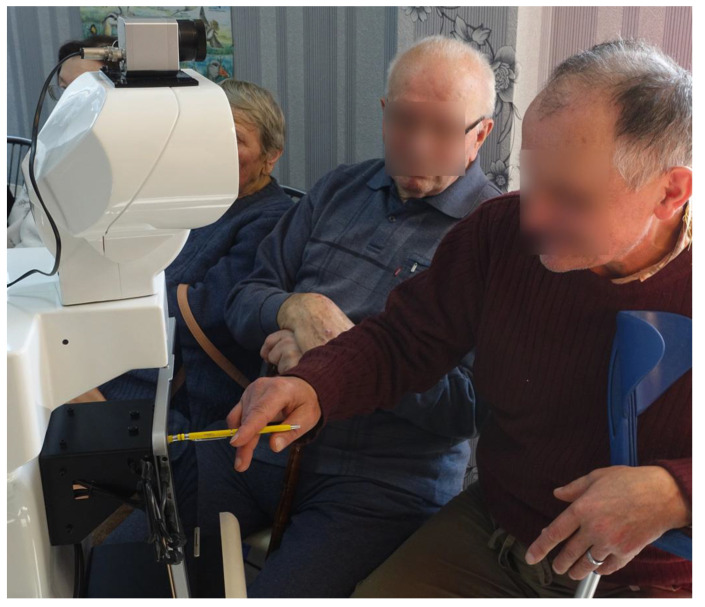
Interaction of older adults with the TIAGo robot (author: Slawomir Tobis).

The purpose of using the GQS in the study was to translate the tool in accordance with the WHO principles for translation and cultural adaptation of healthcare questionnaires [17] and then to use it in practice in research involving a presentation of the TIAGo robot (Figure 1).

As for the translation, in the first stage, two people (an expert in the field of technology and an expert in health sciences) independently translated the English-language version of the questionnaire into Polish. The differences between the two versions of the translations were discussed by a two-person team, resulting in the draft version of the Polish-language tool. Then, an independent bilingual speaker who did not know the original version of the tool performed the back translation. Again, the same two-person team compared the original version of the questionnaire with the back-translated version and discussed minor differences (synonyms) that were detected in the back translation. The final Polish-language version of the questionnaire was used in the preliminary study of five older subjects to check its comprehensibility. No comments were made.

The GQS consists of five-point semantic differentials about the robot, assembled in five series. The first series deals with Anthropomorphism, the second with Animacy, the third with Likeability, the fourth with Perceived intelligence and the fifth with Perceived safety. The terms used are contradictory, and the subject is asked to rate each category on a scale of 1–5, e.g., unintelligent (1) vs. intelligent (5) (from the Perceived intelligence series) or dead (1) vs. alive (5) from the Animacy series. The Polish version of the questionnaire, translated by us, is available alongside other language versions on the web page of the tool [18].

### 2.2. Statistical Analysis

Statistical analysis was performed using STATISTICA software version 13.1 (StatSoft, Kraków, Poland). The results are presented as means and standard deviations as well as medians due to the lack of normal distribution of some variables, verified with the Shapiro–Wilk test. For subjects fulfilling a condition, percentage values are also used.

Internal consistency was assessed for the entire questionnaire and its individual series using Cronbach’s Alpha coefficient. For the interpretation of results, the George and Mallery rating score was used (≥0.9: excellent, ≥0.8–<0.9: good, ≥0.7–<0.8: acceptable, ≥0.6–<0.7: questionable, ≥0.5–<0.6: poor and <0.5: unacceptable [19].

Results were compared with the Mann–Whitney test in the case of two data sets and with paired ANOVA (post hoc Dunn’s test)—in the case of more than two data sets.

The value of *p* < 0.05 was considered statistically significant.

## 3. Results

### 3.1. Characteristics of the Study Group

The perception of the TIAGo robot by 178 older people, whose average age was 75.2 ± 9.6 years (mean ± SD; median 75 years), but as many as 62 respondents were at least 80 years old (34.8%), was analysed. Among the respondents, there were 103 women (57.9%) and 49 people living in their own homes (27.5%). The largest group of respondents had secondary education (37 people—41.6%), but only 20 (11.2%) had higher education.

The average value of self-assessment of fitness was 3.2.1 ± 1.1 (median 3.0, on a scale of 1–5). In terms of the ease of use of technology, as many as 46 people reported that it was not easy (answer 1 on a scale of 1–5), but 52 people (29.2%) reported that they had no problems with using new technological devices at all. The average result of declared the ease of use of technology was 3.1 ± 1.6 (median 3.0).

### 3.2. Internal Consistency of the Polish Version of the Godspeed Questionnaire Series

Good internal consistency of the Polish version of the entire tool was found: Cronbach’s Alpha coefficient was 0.90. The results for the individual series ranged from 0.75 to 0.94 (Table 1).

### 3.3. Characteristics of the Results Obtained Using the Godspeed Questionnaire Series

**Figure 2:**
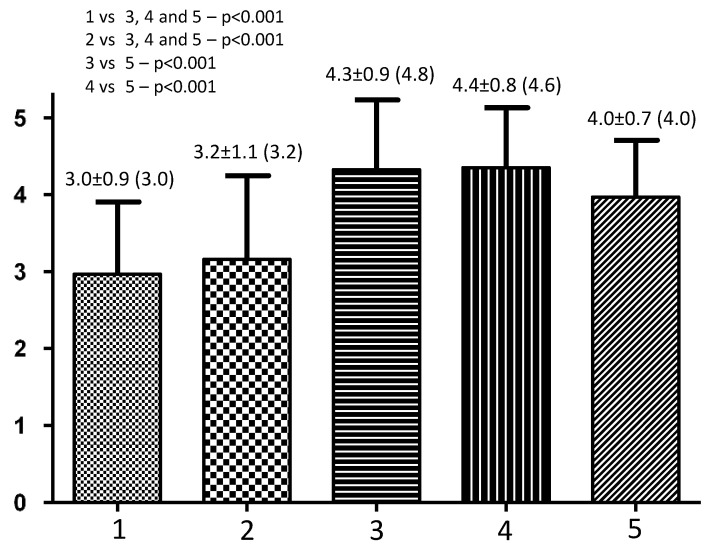
Results of Godspeed Questionnaire Series (*n* = 178 older individuals) based on interaction with the TIAGo humanoid social robot (Series: 1—anthropomorphism, 2—animacy, 3—likeability, 4—perceived intelligence and 5—perceived safety), presented as mean ± SD (median).

The mean scores for series 1 (Anthropomorphism) and series 2 (Animacy) were comparable but lower than all other series (*p* < 0.001). In turn, the average scores for series 3 and 4 (Likeability and Perceived intelligence) did not differ from each other but were higher in relation to the average results of series 5 (Perceived safety—*p* < 0.001). Detailed data are presented in Figure 2.

It was found that age, gender and education did not influence the answers given about the TIAGo robot in any of the GQS series, as did declarations of ease of use of technology and self-assessment of independence. Solely the place of residence influenced the results of the Likeability and Perceived safety series; in both series, people living in institutions scored higher (Table 2).

## 4. Discussion

The assessment of the robot’s perception is an essential element of studying human–robot interaction. One of the recommended assessment tools is the Godspeed Questionnaire Series. According to the suggestion of its author—even if the respondents speak English, one should use the version in the native language to fully optimise the accessibility of the questionnaire [14]. Henceforth, the lack of a Polish version of the tool motivated us to start our study. We reported our activities to the author of the tool, and thanks to that, the version created by us is now on the official list of available languages [18]. While performing a current literature review, we came across another Polish version of GQS [20]. This is, however, not a direct translation of the original tool since it uses four (instead of five) series—Animacy and Anthropomorphism have been blended together. Moreover, the individual series are rated, unlike the original, on a seven-point Likert scale, which makes comparisons of results obtained with this variation of the tool difficult. The author of the original tool draws attention to its modifications, including, e.g., other evaluation systems or the transfer of semantic differential scales used in GQS to Likert-type scales [21,22]. The latter change may be counter-productive in regard to bias because, in measuring positive psychological constructs, a semantic differential format has been shown to effectively reduce acquiescence bias without lowering psychometric quality [23].

We prepared the Polish version of GQS according to the WHO rules and found that the internal consistency of the entire tool was excellent and that for all series, Cronbach’s Alpha coefficient decidedly exceeded 0.7. These values do not differ from those shown by Stroessner in the literature review summarising the available GQS applications [24]. It is, however, worth noting that some authors reported the Cronbach’s Alpha for the Perceived safety series obtained by them as lower than 0.7 (i.e., below the “acceptable” threshold) [25,26]. Our value was the lowest of all questionnaire series studied (0.75, on par with anthropomorphism); still, it was above the cut-off point. Therefore, the Polish-language version of the tool has a confirmed internal consistency. This fact is important because—as the author of the tool himself emphasises—although many language versions of the GQS are available, some have not been subjected to any psychometric assessment [14].

In further research, additional attention should be paid to the assessment of the ceiling effect in relation to the perception of the TIAGo robot, as for all items of the Likeability and Perceived intelligence series, we noted a median of five (which means that more than half of the participants gave the highest possible score). Indeed, Piasek and Wieczorowska-Tobis showed, in a study involving five older people exposed to the TIAGo robot for ten weeks in their own homes (within the ENRICHME project), a ceiling effect was observed for the Likeability series because all subjects scored the robot maximally in this series [27]. On the contrary, the author of the questionnaire, based on the analysis of research with the NAO robot [14], draws attention primarily to the risk of a floor effect, i.e., the fact that the tool may not be sensitive enough to detect small variations of low scores for subjects who had a negative opinion on a particular aspect of the robot. We did not notice this issue due to the very good acceptance of the TIAGo robot, which was also observed in our previous studies of older subjects using the UNRAQ (Users’ Needs, Requirements and Abilities Questionnaire) [16,28].

In our current study, the results of the Anthropomorphism and Animacy series for the TIAGo robot were significantly lower than those of the other series. These findings are in line with the results obtained by Schulz et al. [26]. In the previously mentioned study by Piasek and Wieczorowska-Tobis [27], also using the TIAGo robot and the preliminary GQS version, the Anthropomorphism and Animacy series were rated lower than the others. It suggests that concepts related to social presence were not recognised by the participants while interacting with the robot, which might further mean that the robot was not expected to replace the family or friends in the lives of older people. Despite that, the participants perceived the system as useful and likeable, with a maximum score. This contradicts the suggestion of Weiss and Bartneck, based on their study using the NAO robot, that high scores of Anthropomorphism were a predictor for increased ratings of social acceptance [29]. Hence, the results should be related to a specific robot, and in this aspect, the very good acceptance of the TIAGo robot by older adults needs to be emphasised. On the other hand, based on the experience with the NAO robot, the type of interaction scenario is considered the most important for ratings on the Likeability scale [29]. Therefore, the high scores in the Likeability series may have resulted from the wide range of functions of the TIAGo robot, which the participants could use during the interaction. Its relatively low ratings in the Anthropomorphism and Animacy series may be important for considering the uncanny valley effect (UVE). Importantly, Tu et al. stated that the UVE was found in younger and middle-aged adults, and older adults did not present the UVE [30]. On the other hand, the study of Bradwell et al., involving subjects of various ages, suggested that anthropomorphic or biomorphic design enhanced the social presence of socially assistive robots; the only robot lacking such features delivered no evidence for Perceived sociability [31]. Furthermore, Blut et al. stated in their meta-analysis that anthropomorphism was found to be a positive driver of intention to use a robot [32].

To the best of our knowledge, we are the first to compare subjects living in the community and institutions in terms of the direct impression that participants obtain of a specific robot. In our study, the place of residence was the most important factor for the ratings of the TIAGo robot, i.e., we observed differences in the scores awarded between people living in long-term care institutions and community-dwelling for the Likeability and Perceived safety series: the former scored higher. The need for contact with others in institutionalised people may be important here because, for older people living in long-term care settings, loneliness is a common phenomenon, with multiple studies finding links to depression [33]. Actually, using cluster analysis, we were able to distinguish a group of older people in Polish care institutions with relatively good independence in terms of activities of daily living, whose main problem was depressed mood [34]. These individuals may score higher in the Likeability series and, consequently, also in the Perceived safety series. Indeed, as shown in [33], interventions from a social robot may help to tackle loneliness by acting as a direct companion. This contradicts the results obtained by Li et al. [35], who showed that permanent loneliness (that is not easily relieved) negatively influenced participants’ anthropomorphic inclinations and acceptance of the self-designed Social Robot Prototype. This was true of all its considered forms: a picture of the robot, an on-site robot or direct interaction with the robot. This observation suggests that humanness, traditionally considered in interpersonal contexts and involving secondary emotions, is vital also in establishing connections with social robots. Additionally, cultural differences may be important here because, as Dang and Liu showed, Chinese lonely people were less likely to anthropomorphise robots than their American counterparts (Study 1) and showed less preference for anthropomorphic robots (Study 2) [36]; these effects were characteristic of Chinese culture but did not exist in the American context. Our research clearly shows that this was not true for the TIAGo robot. Hence, again, the relationship between research results and the type of robot used should be highlighted (which is important for HRI); such facts are fundamental from the designers’ perspective.

Our study is not without limitations. The most important one is its cross-sectional nature and one-time interaction with the robot. However, it should be emphasised that some HRI studies, including those using GQS, do not include interactions at all, and sometimes the robot is shown in, for example, a video [22,29,35,37,38,39]. Moreover, Schulz et al., who studied the velocity profiles of a moving Fetch robot, stated that people preferred to interact with the robot instead of only watching it move [26]. Therefore, the model we used has clear advantages versus those without practical interaction. Another limitation of the study model results from basing the validation of the questionnaire on its internal consistency. Nevertheless, as the author of GQS pointed out, there is still no gold standard tool to which it could be related [29]. It is also difficult to demonstrate a test–retest evaluation in a reliable manner. Performing a second assessment after the second exposure could exhibit differences in assessment due to the instability of the situation in which the assessment is carried out. In turn, the second assessment, carried out days after the interaction without further exposure to the robot, would refer to a memorised image; its clarity may be affected by the passage of time, so it is difficult to perform a test–retest analysis in these conditions.

## 5. Conclusions

Our study demonstrated good internal consistency of the Polish version of GQS prepared by us: its construction is analogous to other language versions. Hence, its use yields data that can be compared with the results of other authors. However, in the case of HRI, one must bear in mind that the results obtained refer to a specific robot and experimental model (robot in a video, on a still image, on-site, presented without or with interaction), which makes comparisons of results difficult anyway.

In our study, we used the Polish version of GQS to evaluate the TIAGo robot. We found the highest scores for the Likeability and Perceived Intelligence series and the lowest for the Anthropomorphism and Animacy series. This is an important clue for designers that social acceptance does not necessarily go hand in hand with the perception of anthropomorphism and animacy for, at least, some humanoid social robots. The results of our tests differ in many aspects from those obtained using the NAO robot. Therefore, we would like to point out that generalising the scores from tests performed with a specific robot may not be optimal for the solutions to be created. This also indicates the need for thorough development of experimental models because changes in the type of interaction can also have a significant impact on the perception and, thus, the acceptance of robots in care.

## Figures and Tables

**Table 1:** Cronbach’s Alpha coefficient for the Godspeed Questionnaire Series for older adults after interacting with the TIAGo robot (*n* = 178).

Series	Cronbach’s Alpha Coefficient
Anthropomorphism	0.75
Animacy	0.87
Likeability	0.94
Perceived intelligence	0.90
Perceived safety	0.75

**Table 2:** Results of the QS questionnaire distinguished by the analysed parameters (*n* = 178 older individuals; Series: 1—anthropomorphism, 2—animacy, 3—likeability, 4—perceived intelligence and 5—Perceived safety), presented as mean ± SD (median).

		Series 1	Series 2	Series 3	Series 4	Series 5
Age (years)	60–79 (*n* = 118)	2.9 ± 0.9 (2.8)	3.1 ± 1.0 (3.0)	4.3 ± 0.9 (4.6)	4.3 ± 0.8 (4.6)	4.0 ± 0.7 (4.0)
	≥80 (*n* = 60)	3.1 ± 1.0 (3.0)	3.3 ± 1.2 (3.3)	4.4 ± 0.9 (5.0)	4.4 ± 0.8 (4.8)	4.0 ± 0.8 (4.0)
Gender	Females (*n* = 103)	3.0 ± 1.0 (3.0)	3.3 ± 1.2 (3.3)	4.3 ± 0.9 (4.8)	4.3 ± 0.8 (4.6)	3.9 ± 0.8 (4.6)
	Males (*n* = 75)	2.9 ± 0.9 (2.8)	3.4 ± 1.1 (3.4)	4.4 ± 0.9 (4.8)	4.4 ± 0.7 (4.8)	4.0 ± 0.6 (4.2)
Education	Less than secondary (*n* = 89)	3.1 ± 0.9 (3.0)	3.3 ± 1.0 (3.2)	4.4 ± 0.8 (4.8)	4.4 ± 0.8 (4.8)	4.0 ± 0.7 (4.0)
	At least secondary (*n* = 89)	2.9 ± 1.0 (2.8)	3.1 ± 1.1 (3.0)	4.3 ± 0.9 (4.6)	4.3 ± 0.9 (4.6)	4.0 ± 0.8 (4.0)
Place	Community (*n* = 59)	2.8 ± 0.9 (2.7)	3.2 ± 1.0 (3.2)	4.1 ± 1.0 (4.3)	4.2 ± 0.8 (4.4)	3.6 ± 0.8 (3.7)
of living	Institution (*n* = 119)	3.0 ± 1.0 (3.0)	3.4 ± 1.2 (3.4)	4.4 ± 0.9 (4.8) *p* < 0.05	4.4 ± 0.8 (4.8)	4.1 ± 0.7 (4.2) *p* < 0.001
Ease of use	Below average (*n* = 110)	3.1 ± 0.9 (3.0)	3.1 ± 1.1 (2.9)	4.3 ± 1.0 (4.8)	4.3 ± 0.9 (4.8)	3.9 ± 0.8 (4.1)
of technology	Average and above (*n* = 68)	2.9 ± 1.0 (2.8)	3.2 ± 1.1 (3.2)	4.3 ± 0.9 (4.8)	4.4 ± 0.7 (4.6)	4.0 ± 0.7 (4.0)

## Data Availability

The data presented in this study are available from the corresponding author upon reasonable request.
